# Cervical Net: A Novel Cervical Cancer Classification Using Feature Fusion

**DOI:** 10.3390/bioengineering9100578

**Published:** 2022-10-19

**Authors:** Hiam Alquran, Mohammed Alsalatie, Wan Azani Mustafa, Rabah Al Abdi, Ahmad Rasdan Ismail

**Affiliations:** 1 Department of Biomedical Systems and Informatics Engineering, Yarmouk University, Irbid 21163, Jordan; 2 Department of Biomedical Engineering, Jordan University of Science and Technology, Irbid 21163, Jordan; 3 The Institute of Biomedical Technology, King Hussein Medical Center, Royal Jordanian Medical Service, Amman 11855, Jordan; 4 Faculty of Electrical Engineering & Technology, Campus Pauh Putra, Universiti Malaysia Perlis, Arau 02000, Perlis, Malaysia; 5 Advanced Computing, Centre of Excellence (CoE), Universiti Malaysia Perlis (UniMAP), Arau 02000, Perlis, Malaysia; 6 Mechanical Engineering Department, Faculty of Engineering, Universiti Teknologi PETRONAS, Seri Iskandar 32610, Perak, Malaysia

**Keywords:** pap smear, cervical net, shuffle net, canonical correlation analysis (CCA), support vector machine (SVM), random forest (RF), k-nearest neighbour (KNN), artificial neural network (ANN)

## Abstract

Cervical cancer, a common chronic disease, is one of the most prevalent and curable cancers among women. Pap smear images are a popular technique for screening cervical cancer. This study proposes a computer-aided diagnosis for cervical cancer utilizing the novel Cervical Net deep learning (DL) structures and feature fusion with Shuffle Net structural features. Image acquisition and enhancement, feature extraction and selection, as well as classification are the main steps in our cervical cancer screening system. Automated features are extracted using pre-trained convolutional neural networks (CNN) fused with a novel Cervical Net structure in which 544 resultant features are obtained. To minimize dimensionality and select the most important features, principal component analysis (PCA) is used as well as canonical correlation analysis (CCA) to obtain the best discriminant features for five classes of Pap smear images. Here, five different machine learning (ML) algorithms are fed into these features. The proposed strategy achieved the best accuracy ever obtained using a support vector machine (SVM), in which fused features between Cervical Net and Shuffle Net is 99.1% for all classes.

## 1. Introduction

According to the World Health Organization (WHO), cervical cancer is the fourth most common cancer among women globally, with an estimated 604,000 new cases and 342,000 deaths in 2020. About 90% of the new cases and deaths in 2020 occurred in low- and middle-income countries worldwide [1,2]. Cervical cancer begins with no overt signs and has a long latent period, making early detection through regular checkups important. Cancer is a disease in which the body’s cells grow rapidly, generally termed after the part where it originates, even if it spreads to other parts of the body [3,4,5]. Cervical cancer denotes cancer that begins in the cervix [6,7]. In the year 2018, an estimation of more than 500,000 women worldwide were diagnosed with cervical cancer, resulting in approximately more than 300,000 women dying due to cancer. Infection with high-risk human papillomaviruses (HPV), an immensely prevalent virus spread via sexual contact, is associated with almost all cervical cancer cases (99%). Therefore, cervical cancer may be prevented via screening tests and getting a vaccine that defends against HPV infection. In addition, cervical cancer is usually detected with a Pap smear test. It is a painless, fast screening test for precancer or cancer of the uterine cervix. Moreover, the regular Pap test system lowers the cervical cancer incidence rate [8,9,10,11].

Cervical cancer is a fatal condition of which individuals who possess a low level of awareness. Thus, although it is a life-threatening condition, early diagnosis and treatment may assist in its prevention [12]. Nevertheless, most nations lack efficient screening techniques to encounter this kind of cancer. Hence, in this study we provide a comparison of performance indicators. For example, in terms of accuracy, several machine learning (ML) and deep learning (DL) models for cancerous and normal cervical cells were categorised, including their subtypes. The following literature reviews are related to prior studies on classifying cervical cancer cells.

## 2. Review of Study

In 2015, Mbaga et al. [13] explained cervical cancer detection classification utilising a support vector machine (SVM) classifier gaining around 92.961% accuracy. Furthermore, Win et al. [14] suggested a technique for computer-assisted screening of Pap smear images utilising digital image processing. They utilised texture, shape, and colour features to classify Pap smear images with an accuracy of 94.09%. An investigation by Plissiti et al. [15] found a new method for cervical cancer detection using handcrafted cell features and deep learning (DL) features utilising multi-layer perceptron (MLP) and an SVM classifier, which resulted in the best accuracy obtained, 95.35%. On the other hand, Basak et al. [16] found that a fully automated framework that employs feature selection and DL utilising evolutionary optimisation for cytology image classification obtains an accuracy of 97.87%. With the same objective of recognising cervical cancer’s indications utilising cervicography images, Park et al. [17] examined the performance of two distinct models, DL and ML. Applying the ResNet-50 DL, Random Forest (RF), XGboot (XGB), and SVM and ML models, 4119 cervicography images were identified as negative or positive for cervical cancer by employing square images by omitting the vaginal wall areas. Note that the ResNet-50 model outperformed the average (0.82) of the three ML techniques by 0.15 points (*p* < 0.05). Since this process necessitates segmentation and the acquisition of handcrafted characteristics, a mix of ML and DL techniques is the most efficient. Furthermore, the findings of Tripathi et al. [18] are congruent with the findings of this research. They demonstrated DL classification methods utilising the SIPaKMeD Pap smear image dataset to provide a foundation for new classification strategies. The ResNet-152 architecture achieved the greatest classification accuracy of 94.89% utilising this technique.

Alternatively, Al Mubarak et al. [19] used a hybrid, fusion-based, localised imaging and DL technique to categorise squamous epithelium into cervical intraepithelial neoplasia (CIN) grades, utilising a dataset of 83 digitised histology images. For each segment, 27 handmade image features and a rectangular patch comprising sliding window-based convolutional neural network (CNN) features were computed after partitioning the epithelium region into ten vertical segments. Meanwhile, the DL and imaging patch characteristics are merged and utilised as inputs to a secondary classifier for the individual segment and total epithelium classification. With an accuracy of 80.72% in terms of the whole epithelium CIN classification, the hybrid technique outperformed the imaging and DL techniques alone by 15.51% and 11.66%, respectively. On the other hand, Alyafeai and Ghouti [20] discovered variances, proposing that the suggested pipeline comprises two pre-trained DL models for cervix identification and cervical tumour categorisation. The first model discovers the cervix region 1000 times quicker compared to current data-driven algorithms, with a detection accuracy of 0.68 with respect to the intersection of the union (IoU) scale. The second model utilises self-extracted characteristics to categorise cervical cancers. Here, two lightweight models relying on CNN are employed to learn these characteristics. Moreover, the suggested DL classifier outshines prior models in terms of speed and classification accuracy. The area under the curve (AUC) score of our classifier is 0.82, classifying every cervical region 20 times more quickly. In the most recent published research, Alquran et al. [21] proposed an automated system to classify cervical cancer into seven classes on the Harvel dataset. Their approach exploited the benefits of DL with a model of a cascading SVM classifier to achieve the highest accuracy among all previous studies working on a similar dataset, namely, up to 92% for seven classes. Moreover, their method is fast because the image preprocessing step is skipped. 

Missed diagnoses and misdiagnoses often occur due to the high similarity in pathological cervical images, the large number of readings, the long reading time, and the insufficient experience levels of pathologists. In addition, existing models have insufficient feature extraction and representation capabilities, and they suffer from insufficient pathological classification. In 2021, Park et al. [17] mentioned the significant differences between two different models, ML and DL, in identifying signs of cervical cancer using cervicography images. They concluded that the ResNet-50 DL algorithm could perform better than current ML models in identifying cervical cancer using cervicography images. This is supported by Dhawan et al.’s [22] study, which reveals improved techniques for cervical cancer predictive models based on DL and transfer learning techniques. They classify the cervix images into three classes (Type1/Type2/Type3) by creating a Con-vet structure from combinations between pretrained models (InceptionV3, ResNet-50, and VGG19) were used to create ConvNet that can classify the cervix images. The result of the experiment revealed that the InceptionV3 model performs better than VGG19 and ResNet-50, with an accuracy of 96.1% on the cervical cancer dataset. 

In another study, Huang et al. [23] suggest extracting deep convolutional features by fine-tuning pre-trained deep network models, including ResNet-50V2, DenseNet-121, InceptionV3, VGG19 Net, and Inception ResNet, and then local binary patterns and a histogram of the oriented gradient are used to extract traditional image features. The serial fusion effect of the deep features extracted by ResNet-50V2 and DenseNet-121 (C5) is the best, with the average classification accuracy reaching 95.33%, which is 1.07% higher than ResNet-50V2 and 1.05% higher than DenseNet-121. Furthermore, the recognition ability is significantly improved to 90.89%, which is 2.88% higher than ResNet-50V2 and 2.1% higher than DenseNet-121. Thus, this method significantly improves the accuracy and generalisation ability of pathological cervical whole slice image (WSI) recognition by fusing deep features [23]. Mulmule and Kanphade [24] proposed method that employs adaptive fuzzy k-means clustering to separate cell from the unwanted background of the pathological Pap smear image. The 40 features are extracted from the segmented images based on the shape, size, intensity, orientation, colour, energy, and entropy of the nucleus and cytoplasm individually. Finally, the performance of the supervised classification approach utilising an MLP with three kernels and an SVM with five different kernels as the classifiers to predict the cancerous cells is on par with the existing techniques. The classifier is trained and tested on a benchmark database with 280 Pap smear images. Furthermore, the performance of these two classifiers are evaluated and it is found that the MLP classifier with hyperbolic tangent activation function outperforms the SVM classifier in all the performance criteria, with a classification accuracy of 97.14%, sensitivity of 98%, specificity of 95%, and positive predictive value (PPV) of 98% [24].

A particular image can be used by computer-aided diagnosis (CAD) systems that are trained using artificial intelligence (AI) algorithms to predict the possibility of cervical cancer, which has been highlighted in several cervical cancer studies. For example, Nikookar et al. [25] found that a cervical cancer predictor model, which incorporates the result of different classification algorithms and ensemble classifiers, is more effective for cervical cancer stages. They investigated different aggregation strategies to find the best formula for the aggregation function. They then evaluated our method using the quality assessment of the digital colposcopies dataset. Our approach, performing with 96% sensitivity and 94% specificity values, yields a significant improvement in the field. It can now be used in a supporting clinical decision-making strategy by providing more reliable information to the clinical decision makers. With the same objective, Yaman and Tuncer [26] performed a comprehensive review to classify cervical cells in Pap smear images based on two datasets, SIPaKMeD and Mendeley Liquid Based Cytology (LBC). The 1000 features selected by neighbourhood component analysis (NCA) were classified with the SVM algorithm. Both five-fold cross-validation and hold-out validation (80:20) have been utilised as validation techniques. The best accuracies for the SIPaKMeD and Mendeley LBC datasets have been computed as 98.26% and 99.47%, respectively. The obtained results illustrate that the proposed exemplar pyramid model successfully diagnoses cervical cancer using Pap smear images [26].

According to literature reviews, cancer detection in the early stages is crucial for the treatment process. Therefore, early diagnosis/detection is essential for the treatment of cervical cancer. Note that the gold standard for diagnosing cervical cancer is the Pap smear test. In recent years, there has been an increasing interest in artificial intelligence approaches in medical imaging, such as ML, DL, and CNN [27]. ML is a good solution to automatically diagnose cervical cancer, and many computer vision/DL-based models have been presented in the literature. However, the morphological changes and their entanglement in the structural sections of the cells is one of the constraints. DL and ML algorithms possess a substantial improvement in the healthcare industry. Furthermore, advances in deep learning have led to the development of neural network algorithms that today rival human performance in vision tasks, such as image classification or segmentation. The translation of these techniques into clinical science has also significantly advanced medical image analysis [28]. Research has shown that machine learning can improve the effectiveness of medical image analysis. Algorithms can be developed and trained to remove image noise, improve quality, and gather image data in greater quantities and at a faster rate than standard techniques [29]. Moreover, these algorithms enhance the consistency and accuracy of cancer diagnoses. They also aid medical practitioners in terms of work complexity, minimising labour time, and prognosis. 

This study aimed to build a highly accurate computer-aided diagnosis model for cervical cancer. We obtained features from pre-trained CNN models utilising Shuffle Net, applying different classifiers to discriminate the Pap smear images. Subsequently, we created our DL model called Cervical Net with a simple and light structure, in which its features are passed to different ML classifiers. The key point of this paper is not only the novel DL model but the fusion features between the DL descriptors from various structures to obtain a high level of accuracy. The remainder of this article is structured as follows: Section 3 is devoted to the materials and methods, Section 4 focuses on the results and discussion, and the last section concludes.

## 3. Materials and Methods

**Figure 1:**
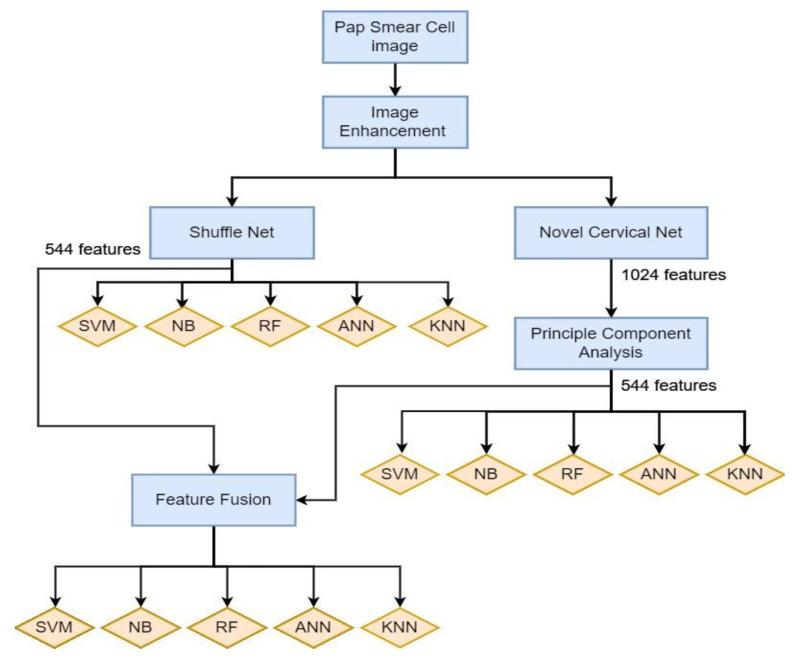
Design of the proposed method.

The proposed method of cervical cytology is displayed in the system flow diagram in Figure 1.

### 3.1. Image Acquisition

**Figure 2:**
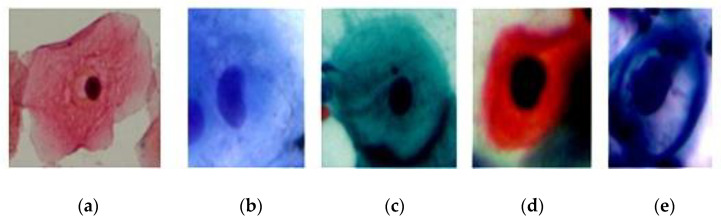
Example images from each class: (**a**) superficial, (**b**) parabasal, (**c**) metaplastic, (**d**) dyskeratotic, (**e**) koilocytotic.

For multi-cell classification, SIPaKMeD datasets were utilised for image acquisition [13]. There were 966 photos in the multi-cell dataset, while 4049 cells were cropped from these images. Note that cells were separated into three stages: normal, benign, and abnormal. Dyskeratotic cells, metaplastic cells, parabasal cells, superficial–intermediate cells, and koilocytotic cells were the five cell types. Table 1 has been created to describe the specifics of each dataset. Table 1 and Figure 2 represent a Pap smear image from the SIPaKMeD dataset.

### 3.2. Image Enhancement

**Figure 3:**
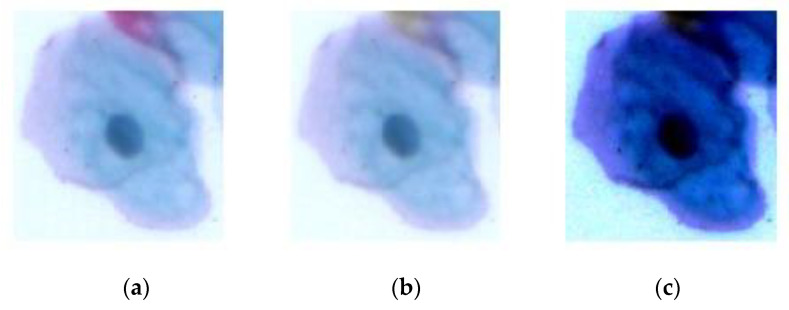
Image enhancement: (**a**) original image, (**b**) noise removal via the median filter, and (**c**) contrast enhancement via histogram equalisation.

As shown in Figure 3a, most Pap smear images were low-contrast and noisy. As a result, image processing was required to reduce noise and raise contrast [30]. To eliminate the noise, a median filter was utilised. The median filter used here is more effective than convolution filters because it removes the noise while preserving the edges. The kernel size in this paper was 3 × 3. Figure 3b shows the image after applying a median filter. Histogram equalisation and normalisation are some of the most common techniques used to enhance the contrast of images, which stretches the histogram of the intensity values into wider ranges. Increasing the contrast leads to extracting more representative features for each class. Figure 3c shows the image after median filtering and histogram equalisation.

### 3.3. Cervical Net

**Figure 4:**
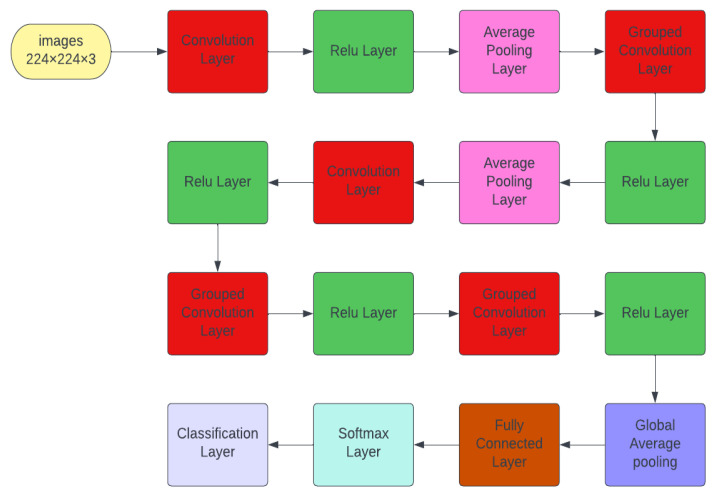
Cervical Net structure.

Cervical Net is a novel DL structure that was designed in this study. Figure 4 shows the layout of its layers with distinguished group convolutional layers. The structure starts with an input layer of an image size of 224 × 224 × 3. Consequently, the coloured image is passed to a convolutional layer with 64 filters, kernel size 7 × 7 and stride 2 × 2. The output is passed to the rectified linear unit (ReLU) layer, which maps the resultant output from the convolutional layer into 1 or -1. To downsample the image feature, it is passed to the average pooling layer with size 3 × 3 and stride 2 × 2. The output is passed to a two-dimensional (2D) grouped convolutional layer, which separates the input into groups and then is applied to slide convolutional filters. The convolution is performed vertically and horizontally, combining the layer of each group independently. In this layer, two groups are used and 94 filters with size 5 × 5 and padding size 2 × 2 × 2 × 2 for all groups. Note that the main goal behind grouping convolutional layers is to obtain higher accuracy than traditional ones. The grouped output is then passed to the ReLU layer and average pooling layer to downsample it with kernel size 3 × 3 and padding 2 × 2. The output is passed to the second convolutional neural network (CNN) for extracting more depth features using 128 filters, kernel size 3 × 3, and padding size 1 × 1 × 1 × 1. Subsequently, the output is passed to the ReLU layer to map it into 1 or −1. The grouped convolutional network is applied to the resultant output with two groups of convolutions using 196 filters, and the kernel size is 3 × 3. The combined output from the depth-wise separable channel is mapped to -1 and 1 using another ReLU function. For extracting depth features and obtaining a higher accuracy, another two groups of the convolutional layer are applied to the mapping output with 128 filters and kernel size 3 × 3. The output is passed to the ReLU layer. The downsampling is performed on the resultant mapping output using the global average pooling layer. The fully connected layer is added to the last output with five neurons compatible with the number of classes, and the softmax layer ends the fully connected layer. This can be defined by the corresponding equation [31,32,33].

$$f\left(x_{i}\right)=\frac{exp\left(x_{i}\right)}{\sum_{j}exp\left(x_{j}\right)},$$

where x refers to the input vector of the layer with size ***K***, denoted by j, in the range of 1:K. Further, $x_{i}$ indicates the *i_th_* individual input. The output of this layer is expressed as probabilities commonly used in multi-classification tasks. Here, the proposed network is terminated by the classification layer. The detailed information regarding the proposed Cervical Net is displayed in Table 2.

### 3.4. Pre-Trained Shuffle Net

Convolutional, pooling and fully linked layers are components of traditional CNN models. The use of large pooling layers and convolution kernels increases the computational complexity of the model. The model’s size and depth increase to enhance the model’s accuracy [34]. Because of the limited performance of some specific applications, the model demands a small size and high accuracy.

**Figure 5:**
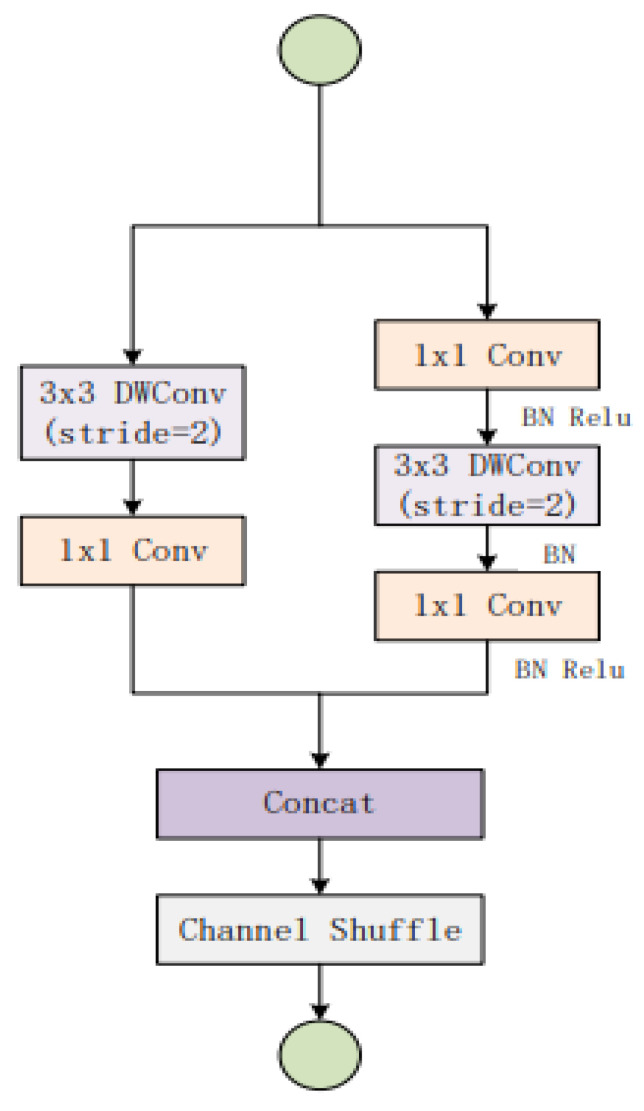
Shuffle Net basic unit [28].

Shuffle Net V2 tackles the aforementioned issues without resorting to large pooling layers or convolution kernels. A depth-wise convolution and a 1 × 1 tiny convolution kernel replace the traditional convolutional layer. Since one convolution kernel is accountable for one input channel with a depth-wise convolution kernel size of 3 × 3, the number of convolution kernels is the same as the number of input channels. To combine characteristics of the depth-wise convolution output, a 1 × 1 convolution is utilised. This increases the network’s expressiveness and nonlinearity without increasing the size of the output feature graph. Furthermore, Shuffle Net downsamples the feature via modifying the depth-wise convolution step instead of utilising the traditional pooling layer [34]. Figure 5 describes the structure of the Shuffle Net basic unit. 

After the convolutional layer, a new layer known as a pooling layer is added. Specifically, after a nonlinearity is employed for the feature map output via a convolutional layer, the pooling layer functions on each feature map independently to construct a new set of pooled feature maps with the same number of characteristics. Moreover, global pooling [35] is another type that occasionally utilises downsamples of the entire feature map to a single value rather than downsampling sections of the input feature map. In our study, we extract features from global pooling and employ them in the classification task.

### 3.5. Deep Features Extraction

Traditional machine learning (ML) algorithms for handcrafted or manual feature extraction have limitations in terms of the correlations and their feature number. With the introduction of artificial intelligence (AI) and deep learning (DL) in the domains of healthcare and the medical sciences, it has become rather common to rely on the findings projected via this decision support system to prevent issues of observer bias. Backpropagation is utilised in DL models to determine the key features, which removes the time-consuming procedure of employing handmade features [36,37].

We utilised both our own structure—Cervical Net—and the pre-trained model to alter the CNN by employing our data, allowing each image to propagate across the layers in a forwarding manner, finishing at the pre-final layer and extracting the output of this layer as the feature vector. Because biological data are inadequate and sparse for DL models to perform effectively if trained from the beginning, we employed pre-learned weights (transfer learning) in this research. For the present study, we have used Cervical Net and Shuffle Net for feature extraction from the model’s global average pooling layer.

### 3.6. Feature Selection

The major goal of utilising a feature selection approach was to determine the crucial features while improving the classifier’s accuracy. Note that the feature selection technique may help ML algorithms train faster by reducing the complexity of the classification model [14]. There are plenty of feature selection algorithms to choose from, and principal component analysis (PCA) is one of them. It is known as a linear dimensionality reduction technique that maximises the variance of the lower dimension into higher dimensional data [16]. PCA is used in this paper to reduce the extracted features of Cervical Net from 1024 to 544 most significant features. 

The number of components in the down-selection stage is chosen based on the number of extracted features from the pre-trained Shuffle Net structure. This procedure is performed using PCA with 95% variance between the selected components.

### 3.7. Feature Fusion

Canonical correlation analysis (CCA) is a standard tool in statistical analysis that measures the linear relationship between two datasets. CCA is an unsupervised representation learning technique for correlating multi-view data by learning a set of projection matrices. The analysis and methods based on CCA are often used in traditional feature fusion methods. It only considers the correlated information of the paired data but ignores the correlated information between the samples in the same class. Furthermore, these methods generally have great deficiencies in exploring the influence of non-negative constraints, feature dimensions, sample size, and noise power. Being complementary to CCA, many discriminant methods have been proposed to extract discriminative features of multi-view data by introducing the supervised class information. However, the learned projection matrices in these methods are mathematically constrained to be of equal rank to the class number and thus cannot represent the original data comprehensively [38]. Canonical correlation analysis (CCA ) considers intraclass and interclass correlations and solves the problem of computation and information redundancy with simple series or parallel feature fusion [39]. Deep CCA based on the encoder–decoder network is designed to extract cross-modal correlations by maximising the relevance between multimodal data [40]. Moreover, CCA is an important method for multiple feature extraction and fusion in which the canonical projective vectors in the classical CCA method satisfy conjugated orthogonality constraints. Class information is useful for CCA, but there is little class information in the scenarios of real applications.

### 3.8. Machine Learning Classifiers 

DL features extracted from Cervical Net are passed to various ML classifiers to obtain the best classifier’s accuracy. The same experiment is performed using the pre-trained Shuffle Net features. The combined features between the novel Cervical Net and Shuffle Net are fused using CCA. The resultant fused features are passed to various ML classifiers to obtain the highest level of accuracy. Subsequently, a comparison is performed between different classifiers for the same features and methods, such as Cervical Net features, Shuffle Net features, or using CCA techniques. 

#### 3.8.1. Support Vector Machine (SVM)

A support vector machine (SVM) refers to a supervised learning model that appropriately labels distinct classes in a set of training samples. The feature plane plot representation of the training data in the SVM model denotes a distinction between the prominent instances representing various classes. A curve that fits in the space between two classes and maintains maximum distances from each class point and SVM can be seen [41,42].

#### 3.8.2. Artificial Neural Networks (ANN)

An artificial neural network (ANN) is a well-known ML technique based on the biological neural network found in the human brain. For example, feedforward neural networks are a typical form of ANN. Once the inputs from neurons are processed in the previous layer, it yields the weight values of each artificial neuron to the proceeding layer. Note that the backpropagation algorithm is the most extensively utilised multi-layer perceptron (MLP) training technique. To reduce inaccuracy, the weights between neurons are altered. Hence, when it comes to learning patterns, this model performs excellently. It can quickly adjust to new data values, but it might be sluggish to converge and runs the risk of a local optimum [43,44].

#### 3.8.3. Naive Bayes

The Naive Bayes technique is a basic probability classifier that calculates probabilities by counting the number of different value and frequency combinations in a dataset. The technique focuses on Bayes’ theorem and assumes that all variables are unaffected by the class variable’s value. Since this conditional independence assumption is hardly valid in real-world applications, it is labelled Naive. Nevertheless, the algorithm learns swiftly in various controlled classification situations [45].

#### 3.8.4. k-Nearest Neighbour (KNN)

Fix and Hodges invented the supervised k-nearest neighbour (KNN) classification technique in 1951 [46], which categorises a data point depending on the class of its neighbours. Moreover, the classification findings are provided depending on the nearest neighbour’s k-value, which was set to 1. Here, the closest k-samples from the training set are chosen to categorise the new sample depending on its attribute vector. As a result, the new vector is directed at it via examining the classes into which the candidate’s samples are categorised [47].

#### 3.8.5. Random Forest (RF)

The random forest (RF) classifier comprises numerous decision trees [48], where every node in the tree contains a set of training cases and a predictor. At each attribute split, a random selection of features is chosen depending on the bagging approach. The trees continue to grow until they attain a certain depth, where a class voting system is established when a large number of trees have been generated [47].

## 4. Results and Discussion

The SIPaKMeD (multi-cell) dataset was utilised to test the efficiency of our suggested method. There was a total of 996 images, with 4049 cells cropped. These cells were categorised into five classes: class 1, superficial–intermediate cells; class 2, parabasal cells; class 3, metaplastic cells; class 4, dyskeratotic cells; and class 5, koilocytotic cells. After processing the images using the convolutional neural network (CNN) architectures, deep features were extracted from global pooling layers. 

### 4.1. Shuffle Net Features 

**Figure 6:**
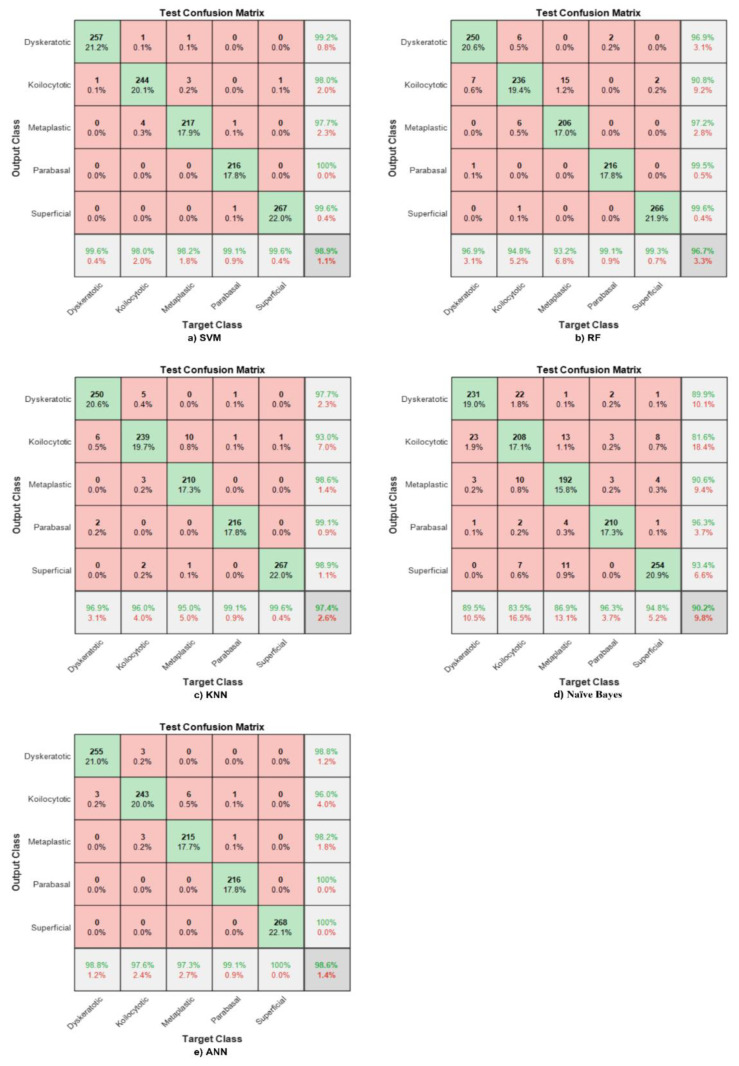
Confusion matrix with respect to Shuffle Net features for different ML classifiers. (**a**) SVM, (**b**) RF, (**c**) KNN, (**d**) Naïve Bays, (**e**) ANN.

Utilising the extracted features from the global pooling layer, different classifiers were used to classify the images into five classes, including support vector machine (SVM), random forest (RF), k-nearest neighbour (KNN), Naive Bayes, and artificial neural network (ANN). At the same time, we utilised 70% of the data as training and 30% as testing. Figure 6 illustrates the confusion matrices result of classifiers, where the test accuracy reaches 98.9%, 96.5%, 97.3%, 89.7%, and 98.7%, respectively, and the training accuracy reaches 100% for all the different classifiers.

In Figure 6a, the diagonal represents the correctly classified observations, whereas the off-diagonal cells indicate incorrectly classified observations. Note that the column on the far right of the plot shows the precision or positive predictive value (PPV). The row at the bottom of the plot refers to the recall or true positive rate (TPR) or sensitivity. Meanwhile, the cell in the bottom right of the plot shows the overall accuracy. The overall accuracy here is 98.9%, and metaplastic benign cells obtain the highest sensitivity and precision of 100%.

The same Shuffle Net features are fed to the RF classifier. The overall accuracy reaches 96.5%, and parabasal malignant cells reach the highest PPV, which is 100%. However, dyskeratotic normal cells obtain the highest sensitivity, which reaches 97.8%, as shown in Figure 6b. 

The accuracy of the hybrid model between Shuffle Net features and KNN does not exceed 97.3%. Meanwhile, the highest precision is in parabasal malignant cells, and the highest recall is in superficial malignant cells, which is represented in Figure 6c. 

Naive Bayes is exploited to classify five cells whose highest accuracy does not exceed 89.7%, and the best sensitivity is obtained by metaplastic benign cells, reaching 95.2%. The parabasal PPV is 99.5%. This is clearly shown in Figure 6d. 

An ANN was used in this study and was fed with Shuffle Net features to obtain the second highest accuracy, reaching 98.7%. Dyskeratotic cells have the highest sensitivity, and parabasal cells the highest precision. This is shown in Figure 6e. 

Previous confusion matrices have shown that the SVM has the highest accuracy for all five classes. Other than that, numerous cervical cell classification models have been developed in the literature using the same datasets. However, this study differs from previous ones in that it focuses on handcrafted features, such as shape, texture, and colour, to classify Pap smear images into five classes.

### 4.2. Novel Cervical Net Features

The proposed network was utilised to extract features from the global average pooling layer, in which the number of extracted features was 1024 graphical features. These features were fed to various machine learning (ML) classifier models to obtain the best model using the novel features. Furthermore, the time taken to extract the features for all test images did not exceed 60 s. The corresponding confusion matrices clarify the test phase for each classifier using novel Cervical Net features. 

**Figure 7:**
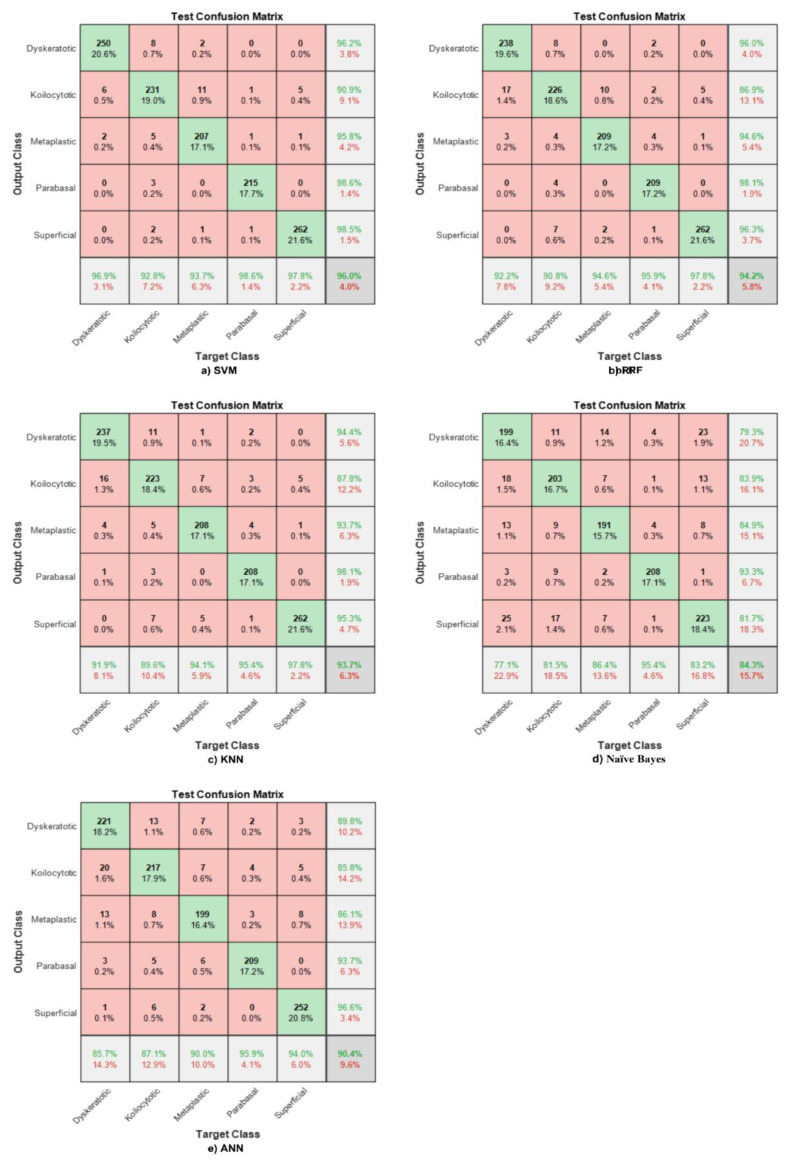
Confusion matrix with respect to Cervical Net features for different ML classifiers. (**a**) SVM, (**b**) RF, (**c**) KNN, (**d**) Naïve Bays, (**e**) ANN.

An SVM was fed with 1024 features to discriminate between various classes. The overall accuracy reached 96%, with higher sensitivity for parabasal normal cells and high precision for parabasal malignant cells. The lowest sensitivity is appeared in malignant cells, namely, dyskeratotic and koilocytotic malignant cells, and it is found in the cells that are very similar in shape and colour, as well. Moreover, the same features were used to design an RF classifier, and the results are clearly shown in Figure 7a. The overall accuracy for the whole system does not exceed 94.2%. The sensitivity of the malignant cells is the lowest in the case of the SVM and the highest in normal cells. Therefore, investigating other methods to enhance the classification process is necessary to discriminate between various classes, either normal or abnormal. 

The KNN classifier was utilised in this study for testing its performance in distinguishing between five classes using the extracted features from Cervical Net. Figure 7b shows that the overall test accuracy is 93.7%. The highest sensitivity was obtained by superficial normal classes. On the other hand, the lowest sensitivity appears in the koilocytotic abnormal class. The highest precision appears in the parabasal normal class, and the lowest PPV is in the koilocytotic abnormal cell. As shown in the KNN confusion matrix, the koilocytotic cell has the lowest TPR and precision. 

In Figure 7, the green color indicates to the correctly classified cells. Furthermore, the red color represents the misclassified cases. 

The lowest accuracy and sensitivity, and even precision, are obtained using the Naive Bayes classifier, in which the accuracy does not exceed 85%. The sensitivity is poor in all classes, as well as the precision. Figure 7c clarifies the confusion matrix generated using test features with the lowest sensitivity appearing among malignant cells. On top of that, the precision of malignant cells is also low. 

An ANN was used in this paper to evaluate the efficiency of the extracted features from the global average pooling from Cervical Net for classifying the five classes. The highest accuracy obtained here does not exceed 90.4% for all classes. Figure 7d describes the test confusion matrix, showing that the two abnormal classes have the lowest sensitivity and PPV. 

Utilising the extracted features from Cervical Net shows that the SVM has the highest accuracy for all five classes and behaves the best among all classifiers. 

### 4.3. Feature Fusion (CCA)

**Figure 8:**
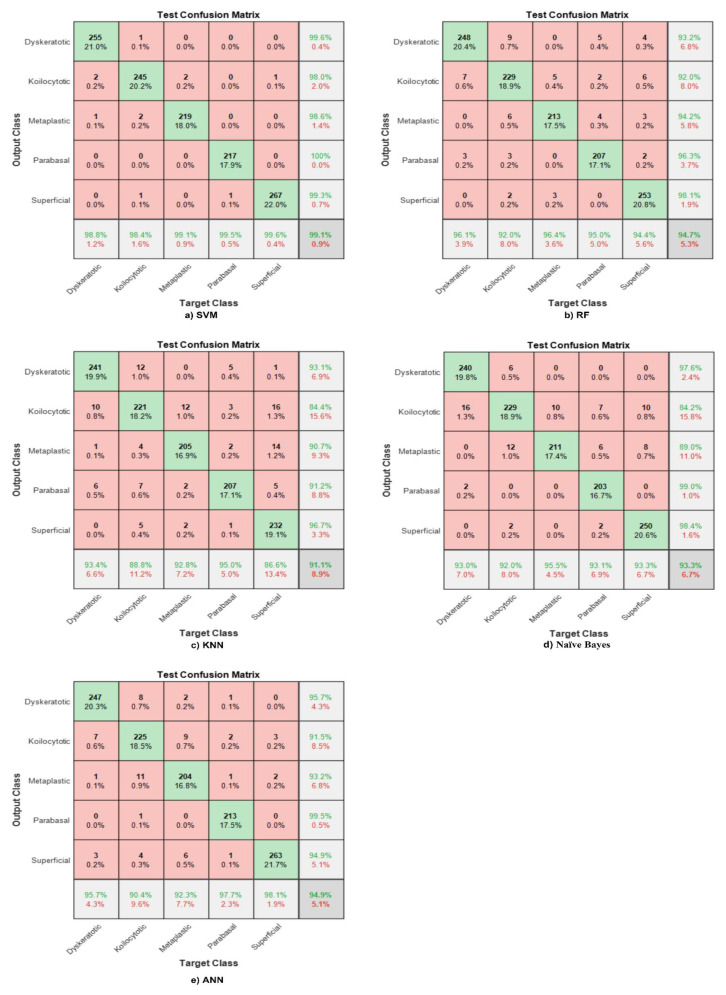
Confusion matrix with respect to CCA features for different ML classifiers. (**a**) SVM, (**b**) RF, (**c**) KNN, (**d**) Naïve Bays, (**e**) ANN.

Feature fusion is a technique used for combining features from various structures, which strengthens the capability of the designed classifier to discriminate between different classes. The extracted features from the global average pooling layer are reduced to 544 features using the principal component analysis (PCA) algorithm to find the most significant features and then combine the resultant descriptors with the graphical features extracted from the global average pooling layer in Shuffle Net. Note that the total number of features after fusion is 544. These features are used to design different ML classifiers. Figure 8 illustrate the confusion matrices for the most known classifiers (SVM, RF, KNN, NB, ANN). Figure 8a shows the maximum accuracy obtained using the SVM’s fusion features, 99.1%. The sensitivity for all classes is elevated to almost 100% for all classes, which helps clinicians in diagnosing even abnormal classes. 

The same features are used to design the RF classifier, and the overall accuracy is 94.7%. The achieved accuracy is higher using the fusion features than the DL descriptors alone. Figure 8b shows that the sensitivity and precision for abnormal classes are enhanced.

The maximum accuracy obtained using fusion features and the KNN classifier is 91.1%, as shown in Figure 8c. Nevertheless, the sensitivity in the normal superficial class is the lowest among all cell types, and abnormal koilocytotic cells have the lowest precision among all classes. 

The same procedure is applied to design the Naive Bayes classifier, and the overall accuracy for the test phase is 93.3%. Although the accuracy is not high, it is better than using DL features solely. Figure 8d shows that the highest recall appears in metaplastic benign cells, which reaches 95.5%, and the highest PPV value appears in koilocytotic abnormal cells. However, the TPR for all abnormal and normal classes exceeds 90%, and the precision is also higher for abnormal and normal classes. 

An ANN classifier was designed with the proposed fusion features, and the accuracy was enhanced to 94.9%, with the best precision obtained is in parabasal normal cells almost 100%. The sensitivity is the best for normal classes discrimination. Note that the sensitivity for all classes exceeds 90%, as shown in Figure 8e. 

**Figure 9:**
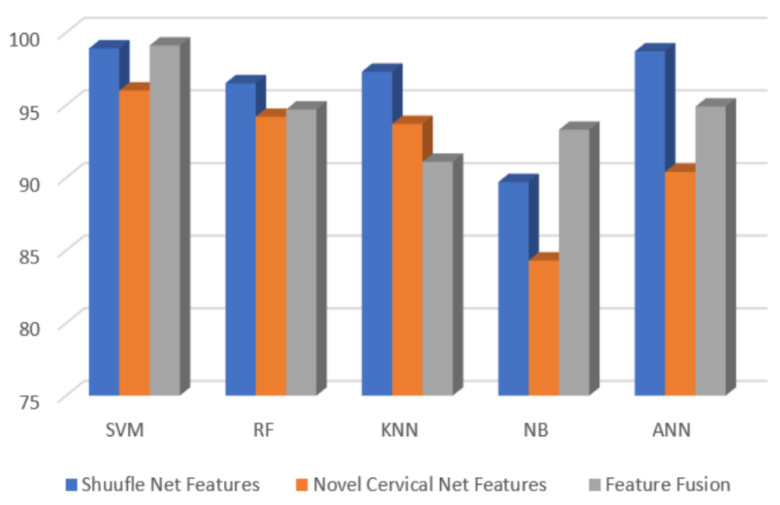
Comparison between various scenarios.

Figure 9 illustrates the accuracies of all the ML classifiers using various scenarios (Shuffle Net features only, novel Cervical Net features only, and the feature fusion from Shuffle Net and Cervical Net). The highest accuracy is obtained by the SVM with feature fusion. 

**Figure 10:**
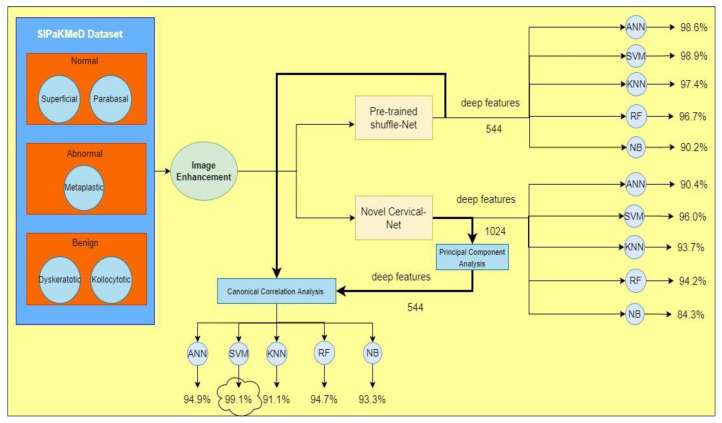
The proposed method with the highest accuracy that has been obtained.

When the proposed method is compared with all previous studies, the obtained results are significant because 99.1% is the highest accuracy achieved using the same dataset. This accuracy was obtained using the CCA method with an SVM classifier. Even though the literature has focused on traditional methods, this study proposed a new structure and utilised the existing method to enhance the resultant accuracy, sensitivity, and precision for all classes. Moreover, the proposed method is fast and accurate. The time needed for testing one new image does not exceed milliseconds, which is acceptable in medical applications, and the proposed structure is simple, unique, and accurate. Table 3 and Figure 10 summarise the results for all the methods.

This method is now compared with previous studies. The proposed method is distinguished from literature due to its simplicity beside to exploiting new features to obtain the highest accuracy for the SIPaKMeD dataset for five classes. Table 4 summarises the results of all previous studies.

The highest accuracy obtained for the same dataset is given in [14]. Nevertheless, this study achieved the highest accuracy in the literature and proposed a novel DL structure that can extract a new feature. Feature engineering is employed here to find the most significant features and combine them with existing features from the pre-trained DL structure. 

## 5. Conclusions

Cervical cancer is the second most frequent cancer among women globally, with a 60% mortality rate. Cervical cancer has no outward symptoms and a long latent period. Therefore, early identification via frequent examinations is critical to counter the high death rate and necessitates using automation in cervical cancer detection. This paper proposed an automated system for cervical cancer using a novel deep learning (DL) structure to extract the features and find the most significant ones. Subsequently, it fused these features with existing pre-trained structures’ graphical descriptors. We suggested a system comprising six steps: image acquisition, image enhancement, feature extraction, feature selection, feature fusion, and classification. This system reached the highest accuracy for five classes at 99.1% in the support vector machine (SVM) classifier after selecting the 544 most significant features from the novel Cervical Net and combining them with 544 from Shuffle Net. The key benefit of our technique is its improved prediction performance in separating classes of Pap smear images and showing better classification accuracy. Furthermore, the obtained result is the best among all previous studies, with the largest dataset for single cells. To summarise, a novel DL structure with modifications to the extracted features can outperform existing machine learning (ML) models when detecting cervical cancer from cervicography images. 

The presented study can be applied in medical fields because it is built based on a huge dataset, making the results more reliable and confidential. Furthermore, this method combines deep learning features and machine learning classifiers, making it easy, fast, and reliable. 

## Figures and Tables

**Table 1:** Specification of five classes of cells obtained from the SIPaKMeD (multi-cell) dataset.

Class	Number of Images	Number of Cells
**Normal Class**		
1. Superficial–Intermediate Cells	126	831
2. Parabasal Cells	108	787
**Benign Cell**		
3. Metaplastic Cells	271	793
**Abnormal Cells**		
4. Dyskeratotic Cells	223	813
5. Koilocytotic Cells	238	825
**Total**	**966**	**4049**

**Table 2:** Structure summaries of Cervical Net.

Layer	Information
Input Layer	Size: 224 × 224 × 3
conv1	Number of Filters: 64
	Kernel Size: 7 × 7
	Stride: 2 × 2
	Padding: 0
Activation Layer	ReLU
Pooling Layer	Type: Average Pooling
	Kernel size: 3 × 3
	Stride: 2 × 2
	Padding: 0
Grouped Convolutional Layer	Number of Groups: 2
	Number of Filters: 94
	Kernel Size: 5 × 5
	Padding: 2 × 2 × 2 × 2
Activation Layer	ReLU
Pooling Layer	Type: Average Pooling
	Kernel Size: 3 × 3
	Stride: 2 × 2
	Padding: 0
Convolutional Layer	Number of Filters: 128
	Kernel Size: 3 × 3
	Padding: (1 × 1 ×1 × 1)
Activation Layer	ReLU
Grouped Convolutional Layer	Number of Groups: 2
	Number of Filters: 192
	Kernel Size: 3 × 3
	Padding: (1 × 1 × 1 × 1)
Activation Layer	ReLU
Grouped Convolutional Layer	Number of Groups: 2
	Number of Filters: 128
	Kernel Size: 3 × 3
	Padding: (1 × 1 × 1 × 1)
Activation Layer	ReLU
Pooling Layer	Type: Global Average Pooling
Fully connected Layer	5 neurons
Softmax Layer	
Classification Layer	

**Table 3:** The results obtained for all proposed methods.

	Shuffle Net	Cervical Net	Feature Fusion (CCA)
**SVM**	98.90%	96.00%	99.10%
**RF**	96.70%	94.20%	94.70%
**KNN**	97.40%	93.70%	91.10%
**Naïve Bayes**	90.20%	84.30%	93.30%
**ANN**	98.60%	90.40%	94.90%

**Table 4:** Comparison of the proposed method with previous studies.

Study	Method	Dataset	Classes	Accuracy
Mbaga et al. [11]	SVM	Herlev dataset	7 classes	92.96%
Win et al. [12]	SVM, KNN, boosted trees, bagged trees, and major voting	SIPaKMeD dataset	2 classes 5 classes	98.27% 94.09%
Plissiti et al. [13]	MLP and SVM	SIPaKMeD dataset	5 classes	95.35%
Basak et al. [14]	feature selection and DL	SIPaKMeD dataset	5 classes	97.87%
Park et al. [15]	ResNet-50 and SVM	Cervicography images	2 classes	82.00%
Tripathi et al. [16]	ResNet-152	SIPaKMeD dataset	5 classes	94.89%
Al Mubarak et al. [17]	Fusion based and CNN		4 classes	80.72%
Alquran et al. [19]	DL and cascading SVM	Herlev dataset	7 classes	Up to 92%
Dhawan et al. [20]	InceptionV3	Kaggle dataset	3 classes	96.10%
Huang et al. [21]	ResNet-50V2 and DenseNet-121	Tissue biopsy image dataset	4 classes	95.33%
Mulmule and Kanphade [22]	MLP with three kernels and SVM	Benchmark database		97.14%
Nikookar et al. [23]	Artificial intelligence	Digital colposcopy dataset	2 classes	96% for sensitivity and 94% for specificity
Yaman and 155 Tuncer [24]	SVM	SIPaKMeD Mendeley	2 classes	98.26% 99.47%
**This study**	**Cervical Net and feature fusion with ML classifiers**	**SIPaKMeD**	**5 classes**	**99.1%**

## Data Availability

The dataset analysed during the current study was derived from the SIPaKMeD database, which consists of 4049 manually isolated pap smear cell images. This dataset has been publicly available online since 2018. It is available on the corresponding website: https://www.cs.uoi.gr/~marina/sipakmed.html (accessed on 15 March 2022).

## References

[1] World Health Organization (2020). WHO Cancer Regional Profile 2020.

[2] Sung H., Ferlay J., Siegel R.L., Laversanne M., Soerjomataram I., Jemal A., Bray F. (2021). Global Cancer Statistics 2020: GLOBOCAN Estimates of Incidence and Mortality Worldwide for 36 Cancers in 185 Countries. CA Cancer J. Clin..

[3] Ferlay J., Colombet M., Soerjomataram I., Mathers C., Parkin D.M., Piñeros M., Znaor A., Bray F. (2019). Estimating the global cancer incidence and mortality in 2018: GLOBOCAN sources and methods. Int. J. Cancer.

[4] Siegel R.L., Miller K.D., Fuchs H.E., Jemal A. (2021). Cancer Statistics, 2021. CA Cancer J. Clin..

[5] Siegel R.L., Miller K.D., Jemal A. (2020). Cancer statistics, 2020. CA Cancer J. Clin..

[6] Mustafa W.A., Halim A., Nasrudin M.W., Rahman K.S.A. (2022). Cervical cancer situation in Malaysia: A systematic literature review. Biocell.

[7] Nahrawi N., Mustafa W.A., Kanafiah S.N.A.M. (2020). Knowledge of Human Papillomavirus ( HPV ) and Cervical Cancer among Malaysia Residents: A Review. Sains Malays..

[8] William W., Ware A., Basaza-Ejiri A.H., Obungoloch J. (2019). A pap-smear analysis tool (PAT) for detection of cervical cancer from pap-smear images. Biomed. Eng. Online.

[9] Nkwabong E., Badjan I.L.B., Sando Z. (2019). Pap smear accuracy for the diagnosis of cervical precancerous lesions. Trop. Doct..

[10] Mustafa W.A., Halim A., Jamlos M.A., Idrus Z.S.S. (2020). A Review: Pap Smear Analysis Based on Image Processing Approach. J. Phys. Conf. Ser..

[11] Mustafa W.A., Halim A., Rahman K.S.A. (2020). A Narrative Review: Classification of Pap Smear Cell Image for Cervical Cancer Diagnosis. Oncologie.

[12] Varalakshmi P., Lakshmi A.A., Swetha R., Rahema M.A. A Comparative Analysis of Machine and Deep Learning Models for Cervical Cancer Classification. Proceedings of the 2021 International Conference on System, Computation, Automation and Networking (ICSCAN).

[13] Mbaga A.H., ZhiJun P. (2015). Pap Smear Images Classification for Early Detection of Cervical Cancer. Int. J. Comput. Appl..

[14] Win K.P., Kitjaidure Y., Hamamoto K., Aung T.M. (2020). Computer-assisted screening for cervical cancer using digital image processing of pap smear images. Appl. Sci..

[15] Plissiti M.E., Dimitrakopoulos P., Sfikas G., Nikou C., Krikoni O., Charchanti A. Sipakmed: A New Dataset for Feature and Image Based Classification of Normal and Pathological Cervical Cells in Pap Smear Images. Proceedings of the International Conference on Image Processing, ICIP.

[16] Basak H., Kundu R., Chakraborty S., Das N. (2021). Cervical Cytology Classification Using PCA and GWO Enhanced Deep Features Selection. SN Comput. Sci..

[17] Park Y.R., Kim Y.J., Ju W., Nam K., Kim S., Kim K.G. (2021). Comparison of machine and deep learning for the classification of cervical cancer based on cervicography images. Sci. Rep..

[18] Tripathi A., Arora A., Bhan A. Classification of cervical cancer using Deep Learning Algorithm. Proceedings of the 5th International Conference on Intelligent Computing and Control Systems, ICICCS 2021.

[19] AlMubarak H.A., Stanley J., Guo P., Long R., Antani S., Thoma G., Zuna R., Frazier S., Stoecker W. (2019). A hybrid deep learning and handcrafted feature approach for cervical cancer digital histology image classification. Int. J. Healthc. Inf. Syst. Inform..

[20] Alyafeai Z., Ghouti L. (2020). A fully-automated deep learning pipeline for cervical cancer classification. Expert Syst. Appl..

[21] Alquran H., Mustafa W.A., Qasmieh I.A., Yacob Y.M., Alsalatie M., Al-Issa Y., Alqudah A.M. (2022). Cervical Cancer Classification Using Combined Machine Learning and Deep Learning Approach. Comput. Mater. Contin..

[22] Dhawan S., Singh K., Arora M. (2021). Cervix image classification for prognosis of cervical cancer using deep neural network with transfer learning. EAI Endorsed Trans. Pervasive Health Technol..

[23] Huang P., Tan X., Chen C., Lv X., Li Y. (2021). AF-SENet: Classification of cancer in cervical tissue pathological images based on fusing deep convolution features. Sensors.

[24] Mulmule P.V., Kanphade R.D. (2021). Supervised classification approach for cervical cancer detection using Pap smear images. Int. J. Med. Eng. Inform..

[25] Nikookar E., Naderi E., Rahnavard A. (2021). Cervical cancer prediction by merging features of different colposcopic images and using ensemble classifier. J. Med. Signals Sens..

[26] Yaman O., Tuncer T. (2022). Exemplar pyramid deep feature extraction based cervical cancer image classification model using pap-smear images. Biomed. Signal Process. Control.

[27] Coppola F., Faggioni L., Gabelloni M., De Vietro F., Mendola V., Cattabriga A., Cocozza M.A., Vara G., Piccinino A., Lo Monaco S. (2021). Human, All Too Human? An All-Around Appraisal of the ‘Artificial Intelligence Revolution’ in Medical Imaging. Front. Psychol..

[28] Wang R., Lei T., Cui R., Zhang B., Meng H., Nandi A.K. (2022). Medical image segmentation using deep learning: A survey. IET Image Process..

[29] Erickson B.J., Korfiatis P., Akkus Z., Kline T.L. (2017). Machine learning for medical imaging. Radiographics.

[30] Mustafa W.A., Sam S., Jamlos M.A., Khairunizam W. (2021). Effect of different filtering techniques on medical and document image. Lect. Notes Electr. Eng..

[31] Alqudah A., Alqudah A.M., Alquran H., Al-zoubi H.R., Al-qodah M., Al-khassaweneh M.A. (2021). Recognition of handwritten arabic and hindi numerals using convolutional neural networks. Appl. Sci..

[32] Alsharif R., Al-Issa Y., Alqudah A.M., Qasmieh I.A., Mustafa W.A., Alquran H. (2021). Pneumonianet: Automated detection and classification of pediatric pneumonia using chest X-ray images and cnn approach. Electronics.

[33] Alawneh K., Alquran H., Alsalatie M., Mustafa W.A., Al-Issa Y., Alqudah A., Badarneh A. (2022). LiverNet: Diagnosis of Liver Tumors in Human CT Images. Appl. Sci..

[34] Liu H., Yao D., Yang J., Li X. (2019). Lightweight convolutional neural network and its application in rolling bearing fault diagnosis under variable working conditions. Sensors.

[35] Brownlee J. (2019). A Gentle Introduction to Pooling Layers for Convolutional Neural Networks. Mach. Learn. Mastery.

[36] Basak H., Kundu R. (2021). Comparative Study of Maturation Profiles of Neural Cells in Different Species with the Help of Computer Vision and Deep Learning. Commun. Comput. Inf. Sci..

[37] Basak H., Ghosal S., Sarkar M., Das M., Chattopadhyay S. Monocular Depth Estimation Using Encoder-Decoder Architecture and Transfer Learning from Single RGB Image. Proceedings of the IEEE 7th Uttar Pradesh Section International Conference on Electrical, Electronics and Computer Engineering (UPCON).

[38] Wang Z., Wang L., Huang H. (2021). Sparse additive discriminant canonical correlation analysis for multiple features fusion. Neurocomputing.

[39] Shi J., Chen C., Liu H., Wang Y., Shu M., Zhu Q. (2021). Automated Atrial Fibrillation Detection Based on Feature Fusion Using Discriminant Canonical Correlation Analysis. Comput. Math. Methods Med..

[40] Zhang K., Li Y., Wang J., Wang Z., Li X. (2021). Feature fusion for multimodal emotion recognition based on deep canonical correlation analysis. IEEE Signal Process. Lett..

[41] Pisner D.A., Schnyer D.M. (2019). Support vector machine. Machine Learning: Methods and Applications to Brain Disorders.

[42] Alquran H., Qasmieh I.A., Alqudah A.M., Alhammouri S., Alawneh E., Abughazaleh A., Hasayen F. The melanoma skin cancer detection and classification using support vector machine. Proceedings of the 2017 IEEE Jordan Conference on Applied Electrical Engineering and Computing Technologies, AEECT 2017.

[43] Haykin S. (2009). Neural Networks and Learning Machines.

[44] Rumelhart D.E., Hinton G.E., Williams R.J. (1986). Learning representations by back-propagating errors. Nature.

[45] Chen S., Webb G.I., Liu L., Ma X. (2020). A novel selective naïve Bayes algorithm. Knowl.-Based Syst..

[46] Fix E., Hodges J.L. (1989). Discriminatory Analysis. Nonparametric Discrimination: Consistency Properties. Int. Stat. Rev. Rev. Int. Stat..

[47] Alquran H., Alsleti M., Alsharif R., Qasmieh I.A., Alqudah A.M., Harun N.H.B. (2021). Employing texture features of chest x-ray images and machine learning in covid-19 detection and classification. Mendel.

[48] Sun G., Li S., Cao Y., Lang F. (2017). Cervical cancer diagnosis based on random forest. Int. J. Perform. Eng..

